# Stability Assessment of Furosemide Oral Suspension in Hospital Extemporaneous Preparations

**DOI:** 10.3390/ph18070937

**Published:** 2025-06-20

**Authors:** Fai Alkathiri, Omamah Eid, Njoud Altuwaijri, Rihaf Alfaraj, Eram K. Eltahir, Hend Alsabbagh, Shamma Bin Shoia, Mashal Aljead, Haya H. Alnufaie, Ghadah AlToum

**Affiliations:** 1 Department of Pharmaceutics, College of Pharmacy, King Saud University, Riyadh 11451, Saudi Arabia; falkathire@ksu.edu.sa (F.A.); 445205574@student.ksu.edu.sa (O.E.); ralfaraj@ksu.edu.sa (R.A.); ekeltahir@ksu.edu.sa (E.K.E.); 442202191@student.ksu.edu.sa (H.A.); 442201725@student.ksu.edu.sa (S.B.S.);; 2 Prince Sultan Military Medical City, Riyadh 11159, Saudi Arabia; maljead@psmmc.med.sa (M.A.); halnufaie@psmmc.med.sa (H.H.A.)

**Keywords:** furosemide, suspensions, extemporaneous, quality control, stability

## Abstract

**Background:** Furosemide is a loop diuretic used extensively to treat adult and pediatric patients. In some hospitals, furosemide oral liquids are not available in stock, thus necessitating the extemporaneous preparation of the drug. This study evaluates the stability of on-the-spot formulations of furosemide oral suspensions from crushed tablets evaluated in various vehicles: Dextrose 50%, Dextrose 70%, Ora-Sweet, and Ora-Plus over 60 days. This examination was prompted by the frequent shortage of certain excipients in the hospital, leading to the need to switch to Dextrose 50% or Dextrose 70% when Ora-Sweet and Ora-Plus are out of stock. **Methods:** The extemporaneous furosemide oral suspensions were prepared following the same compounding method used in the pharmacy. The suspensions were maintained at 4 °C in the refrigerator and assessed immediately and later, on days 7, 14, 30, and 60. The assessed parameters included visual appearance, redispersion time, sedimentation volume, and pH levels for stability analysis. We also examined the drug content, dissolution of the suspension, and microbiological stability. **Results:** Initial examinations indicated that Dextrose 50% and Ora-Plus maintained pH levels and stable appearances, while significant changes, mainly in appearance and redispersion time, indicated the instability of Dextrose 70%. Ora-Sweet showed fluctuations but stabilized by day 30. Dissolution studies demonstrated that Ora-Plus had dissolution characteristics superior to the other formulations, while Dextrose 50% showed declining dissolution percentages over time. Overall, the Ora-Plus vehicle showed superior stability (60 days), followed by Ora-Sweet (30 days), while Dextrose 70% and Dextrose 50% showed shorter stability durations of 14 and 7 days, respectively. The microbiological test results showed no microbial growth. **Conclusions:** This study demonstrates that the vehicle used in extemporaneous furosemide suspensions critically affects their stability and performance. Ora-Plus emerged as the most suitable vehicle, maintaining physical, chemical, and microbiological stability over 60 days, with consistent pH, redispersion, and dissolution behavior. Ora-Sweet showed intermediate stability (30 days), while Dextrose 50% and 70% exhibited early instability—7 and 14 days, respectively—marked by sedimentation, poor redispersibility, and declining drug release. These findings underscore the importance of vehicle selection and regular stability monitoring in compounded formulations to ensure therapeutic reliability and patient safety.

## 1. Introduction

The term “extemporaneous compounding” refers to the act in which pharmacists utilize established compounding techniques to manipulate drugs to prepare appropriate dosage forms in the absence of commercial forms of these drugs [1]. These preparations are generally required to meet the needs of specific patients (e.g., pediatric patients, patients who have difficulty swallowing, or patients with nasogastric feeding tubes) [2]. Pharmacists prepare appropriate formulations by crushing solid tablets or opening capsules, and the resultant powdered tablets or capsule contents are dissolved or suspended in a variety of excipients to obtain a liquid oral formulation, or the process can be reformulated to reduce its strength [3]. However, limited information exists on the stability of extemporaneous preparations. Ensuring stability and compatibility when compounding these preparations is critical because these factors determine their quality, safety, and efficacy [4]. Stability is defined by the United States Pharmacopeia (USP) as “the extent to which a product retains, within specified limits and throughout its period of storage and use, the same properties and characteristics that it possessed at the time of its manufacture” [5]. Because the shelf life of compounded formulations has not been verified by stability tests [6], their original strength and purity cannot be presumed to be maintained over time. Therefore, instead of expiration dates, beyond use dates (BUDs) are used [4]. A BUD refers to a date after which the preparation is not to be used [7]. Alkhatib et al. investigated extemporaneous compounding practices in hospital and community pharmacies throughout the governorates in Jordan [8] and found that although most pharmacies practiced compounding, stability data were notably lacking; for example, the pharmacists’ own experience and physicians’ orders were the primary sources for estimating the expiration dates for compounded drugs. Furthermore, no pharmacy performed quality control tests on these products.

When assigning BUDs, pharmacists may face difficulties attributed to limited support from scientific evidence or pharmacopeias for formulating oral liquids [9]. However, compounded suspensions are typically given short expiration dates, requiring patients to renew their prescriptions regularly, which is inconvenient for patients and leads to higher healthcare costs [10,11]. Nevertheless, the USP has assigned BUDs for compounded nonsterile formulations, for which no USP monograph or stability data are available. For non-preserved aqueous preparations, the assigned BUD is 14 days under refrigeration, whereas for preserved aqueous preparations, the assigned BUD is 35 days under controlled room temperature and refrigeration. For nonaqueous oral preparations and other nonaqueous dosage forms, the assigned BUDs are 3 months and 6 months, respectively [6]. However, extemporaneously compounded formulations should be evaluated in terms of their chemical, physical, and microbiological stability [1].

Many factors can affect the stability of a drug, including temperature, pH, moisture, light, oxygen presence, and the excipients used [12]. In addition, the stability profile may vary depending on the suspending agent used. As a consequence of exchanging the vehicles, the prepared drug may not retain its stability over the expected shelf life, leading to a reduced dose being delivered to the patient [10].

Previous studies have reported the effects of specific vehicles on the stability of extemporaneous preparations. For example, the superior stability of quetiapine oral compounded suspensions was reported when the Ora-Blend vehicle was used compared with the Ora-Sweet vehicle at 4 °C and 22 °C [13].

Furosemide, a diuretic used extensively to treat adult and pediatric patients, has been approved for the treatment of conditions accompanied by fluid retention or edema (e.g., heart failure and renal and hepatic diseases), as well as hypertension. It is a commonly compounded drug for the treatment of cardiovascular conditions in pediatric patients, especially neonates [14]. Currently, furosemide is available on the market in both injection and tablet forms; in some countries, it is also available as an oral syrup [15].

Furosemide is insoluble in water, which makes it challenging to formulate as an aqueous solution. Ethanol is commonly added to commercially available formulations to enhance its solubility in water. Unfortunately, two ingredients in the available syrup (ethanol and propylene glycol) can harm the central nervous system, especially in neonates [16,17,18,19]. To overcome this safety issue and address its lack of availability in many countries, furosemide oral liquid can be formulated extemporaneously using an alcohol-free formula. A recent study evaluating global pediatric drug issues identified furosemide as a commonly unavailable drug [20]. In addition, the study found that furosemide was the most problematic cardiovascular drug in terms of acceptability, dosing safety, and the requirement to modify available adult formulations, which might impact the stability of the active pharmaceutical ingredient.

The stability of furosemide has been studied extensively. Furosemide oral liquid can be formulated extemporaneously using commercially available tablets [17,21,22] or pure powder [19]. A simple method involves diluting furosemide injections with water; however, this method has some limitations, including an unpalatable taste attributed to sodium hydroxide and a lack of preservatives [19]. The stability of furosemide is influenced by the pH of the medium—furosemide is unstable in acidic media because acid-catalyzed hydrolysis in aqueous solutions results in the production of a drug degradant, 4-chloro-5-sulfamoylanthranilic acid. In contrast, furosemide has been found to be highly stable in alkaline media [17].

The current study evaluated the stability of extemporaneous formulations of furosemide oral suspensions of crushed tablets over 60 days in four vehicles: Dextrose 50%, Dextrose 70%, Ora-Sweet, and Ora-Plus. The frequent shortages of certain compounding vehicles in hospitals often necessitate substituting Dextrose 50% or Dextrose 70% for Ora-Sweet and Ora-Plus when the latter two are unavailable. These suspensions were primarily intended for pediatric use, which guided the choice of a 2 mg/mL concentration and the selection of vehicles commonly used in these settings. For this study, the suspensions were maintained at 4 °C in a refrigerator. The samples were assessed immediately after compounding (day 0) and on days 7, 14, 30, and 60. Extemporaneous furosemide oral suspensions were compounded using the same method used for patients at the hospital pharmacy. To analyze the stability of the suspensions, the following parameters were evaluated: visual appearance and organoleptic properties, redispersion time, pH levels, suspension viscosity, particle size, zeta potential, and sedimentation volume. In addition, the drug content, dissolution of suspensions, and microbiological stability were assessed.

## 2. Results

### 2.1. Standard Calibration Curve

**Figure 1:**
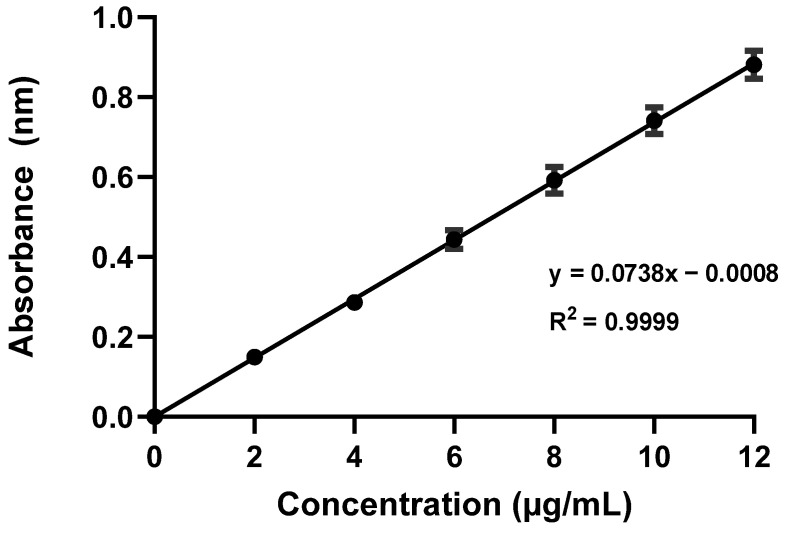
Standard calibration curve of furosemide in 0.1 N NaOH using an ultraviolet–visible spectrophotometer at a wavelength of 270 nm.

A standard calibration curve was obtained by plotting six concentrations of furosemide in µg/mL on the *x*-axis versus the corresponding mean absorbance values on the *y*-axis (Figure 1). Linear regression analysis resulted in a linear equation, y = 0.0738x − 0.0008. The standard curve exhibited a notably high degree of linearity over a concentration range of 2–12 µg/mL with a correlation coefficient (R^2^) of 0.999, which indicated that the method was suitable and applicable for determining furosemide concentrations in different samples. GraphPad was used to calculate the correlation coefficient (R^2^) for the linearity assessment of the calibration curve; an R^2^ value close to 1.00 indicates strong linearity. Furthermore, a highly significant *p*-value of less than 0.0001 was determined via the analysis, further supporting the validity of the calibration.

### 2.2. Visual Appearance and Organoleptic Properties of Furosemide Suspensions

A distinct appearance was noted for all examined suspensions. Dextrose 50%, Dextrose 70%, and Ora-Plus suspensions were white, whereas Ora-Sweet was pale yellow. All suspensions were odorless. No detectable changes in appearance or odor were observed for any suspensions except Dextrose 70%, which developed a thick foam-like appearance by day 7, suggesting physicochemical changes in the formulation (Figure S1).

### 2.3. pH Measurement

An initial reduction in the pH value of the Dextrose 50%-based suspension from 4.57 to 4.11 was observed after 1 week of storage. However, this reduction was followed by a significant elevation in the pH value to 4.67 on day 14, which stabilized around 4.68 after 1 month. Furthermore, the suspension showed a minimal increase to 4.72 on day 60. Overall, this trend shows that Dextrose 50% retains a relatively stable pH after an initial decline, suggesting that it might be an appropriate vehicle for preparations that require a constant pH environment. Conversely, on the day of preparation (day 0), the Dextrose 70%-based suspension had a lower pH of 3.16, which declined further to 2.93 after 1 week. The suspension pH remained relatively stable (3.12 to 3.06) in storage between days 14 to 60. The previous measurements indicated that Dextrose 70% has a high degree of acidity that remains relatively constant over time. This establishes a concern about its suitability for use as a vehicle in drug formulations for which a neutral or mildly acidic environment is preferable, especially in terms of patient acceptability and drug stability. The Ora-Sweet-based suspension provided higher pH stability compared with the Dextrose-based formulations. Slight fluctuations in pH were observed within a narrow range, indicating that Ora-Sweet maintains a stable pH, making it a potentially appropriate vehicle to ensure pH stability over time.

The Ora-Plus-based suspension had superior pH stability compared with Ora-Sweet and all other vehicles examined in this study. Therefore, Ora-Plus might be an appropriate vehicle for drug preparations for which pH consistency is critical. The pH measurements for each vehicle are summarized in Table 1. It was evident that different formulations exhibited different patterns of pH stability and variability.

A two-way analysis of variance (ANOVA) was performed to assess the effects of formulation vehicle and storage time on pH stability across a 60-day period. The results demonstrated a highly significant main effect of formulation vehicle (*p* < 0.001), indicating that the different vehicles exhibited distinct baseline pH characteristics. There was also a significant main effect of storage time (*p* < 0.001), suggesting that the pH values changed appreciably over the course of the study. Importantly, the interaction between the formulation vehicle and storage time was significant as well (*p* < 0.001), revealing that the trends in pH stability varied depending on the specific vehicle used. These findings confirm that both the type of formulation and the duration of storage significantly influence pH stability, and that the pattern of pH change over time is formulation-dependent.

### 2.4. Sedimentation Volume (F)

**Figure 2:**
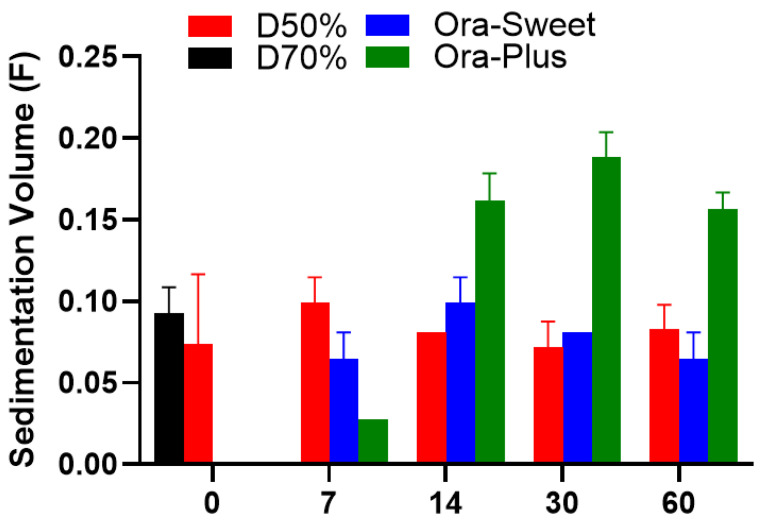
Sedimentation volumes of furosemide suspensions (*n* = 3).

The sedimentation volume (*F*) profiles of the compounded furosemide suspensions over different storage periods are shown in Figure 2. The Dextrose 50% suspension showed a minor elevation in sedimentation on day 7, which stabilized over time. On the day of the preparation, the Ora-Sweet- and Ora-Plus-based suspensions showed no sedimentation, which was attributable to specific ingredients, namely, viscosity enhancers and suspending agents, respectively. Sedimentation was observed in both formulations on day 7. Ora-Sweet had more consistent sedimentation volumes, but Ora-Plus had the highest outright sedimentation volumes. Because of the foam-like appearance exhibited by the Dextrose 70% suspension on day 7, its sedimentation volume was not measured thereafter. Statistical analysis using the Kruskal–Wallis test revealed significant differences among the formulations, with a *p*-value of 0.0356.

### 2.5. Viscosity Measurements (cP)

**Figure 3:**
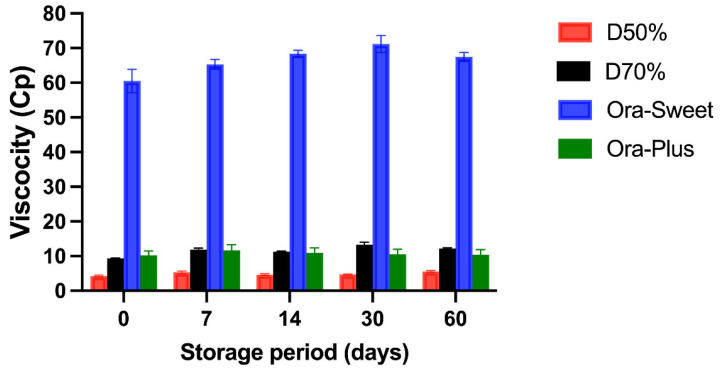
The viscosity of the furosemide suspensions over 60 days at 4 °C (*n* = 3, mean ± standard deviation, *p* > 0.05).

The viscosity (cP) of the four different furosemide suspensions at different storage periods is shown in Figure 3. All suspensions maintained a relatively consistent viscosity. The Ora-Sweet-based suspension had the highest viscosity throughout the study (65–70 cP), and the Dextrose 50% suspension had the lowest viscosity (2–5 cP). The rank order of viscosity was Ora-Sweet > Ora-Plus > Dextrose 70% > Dextrose 50%. A two-way ANOVA was conducted to assess the impact of formulation type and storage duration on sedimentation volume (F). The analysis revealed statistically significant effects for both the formulation (*p* < 0.0001) and time (*p* < 0.0001), as well as a highly significant interaction between the two factors (*p* < 0.0001).

### 2.6. Particle Size Analysis

**Figure 4:**
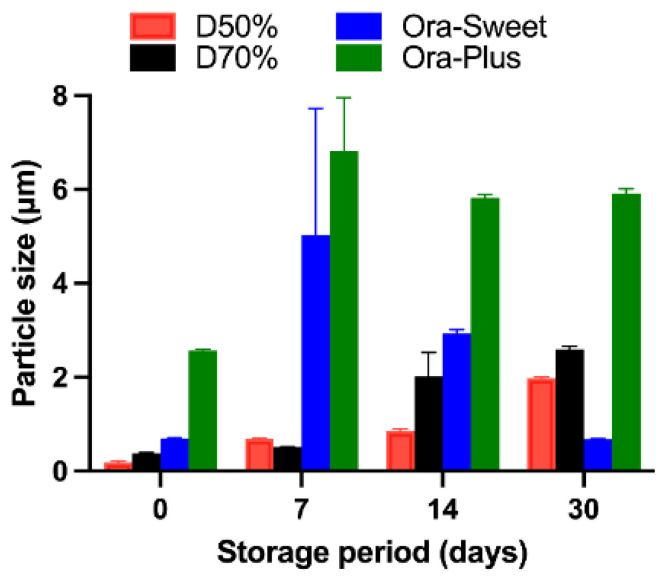
Particle size of furosemide suspensions (*n* = 3) 0–30 days of storage.

The particle size analysis showed a progressive increase over time, with all formulations remaining within measurable limits during the first 30 days (Figure 4). However, after 30 days, significant aggregation was observed—particularly in Ora-Plus and Dextrose 70% vehicles—resulting in particle sizes that exceeded the reliable detection range of the Zetasizer instrument. Therefore, particle size measurements were not conducted beyond day 30. To assess overall changes in particle size across time and between formulations, we performed a two-way ANOVA. The analysis showed significant effects of both formulation type and time (each *p* < 0.0001), as well as a significant interaction, indicating that the particle size growth trends were formulation-dependent.

### 2.7. Zeta Potential

**Figure 5:**
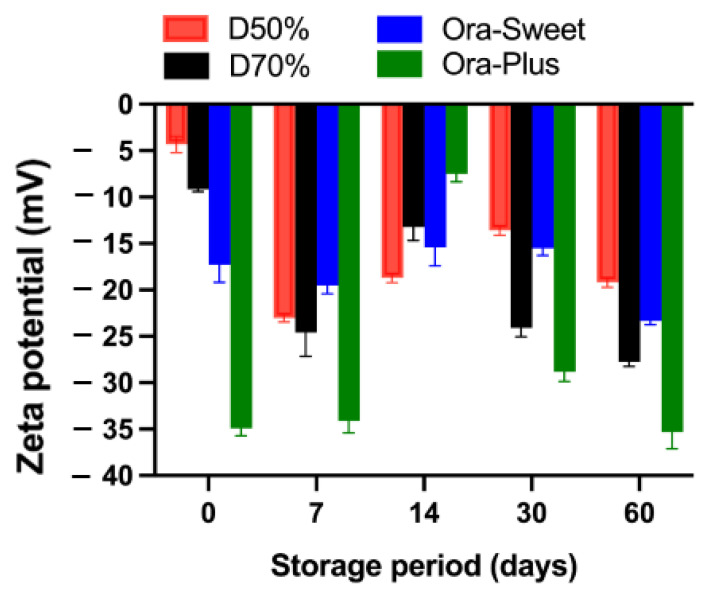
Zeta potential of furosemide suspensions (*n* = 3).

The zeta potential results for the different furosemide suspensions over the study period are presented in Figure 5. All suspensions had negative zeta potential values throughout the study period, with Dextrose 50% exhibiting the lowest negative zeta potential value initially (−4.32 mV), which became more negative over time, whereas Dextrose 70% maintained more negative values at different time points, suggesting better stability and a reduced tendency toward aggregation. Ora-Sweet had more consistent values and a higher degree of stability than the Dextrose-based suspensions. However, in comparison to all other compounded suspensions, Ora-Plus maintained the most negative values, starting at −34.9 mV and reaching −35.3 mV at the end of the study (day 60), suggesting higher electrostatic repulsion, greater particle dispersion, and superior stability compared with the other vehicle formulations.

### 2.8. Content Uniformity Test

**Figure 6:**
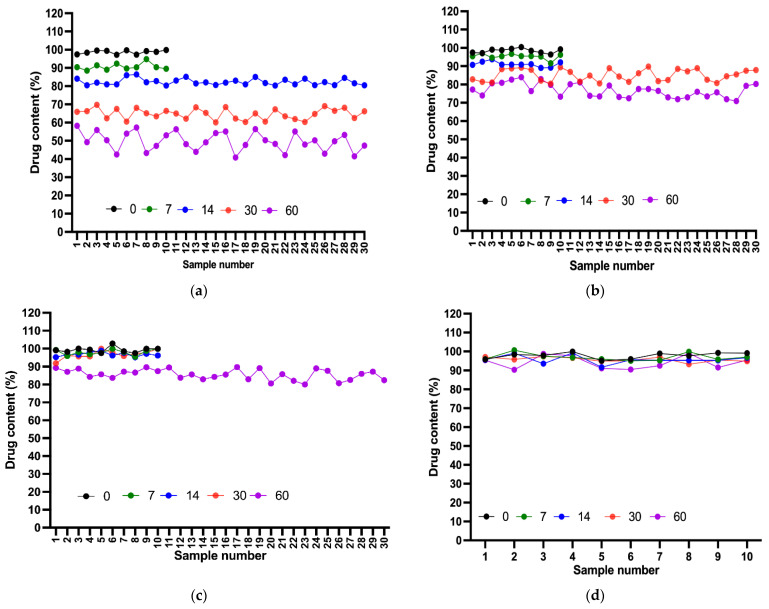
Drug content results for compounded suspensions over a 60-day storage period: (**a**) Dextrose 50%; (**b**) Dextrose 70%; (**c**) Ora-Sweet; (**d**) Ora-Plus.

Various samples of each compounded suspension were assessed to ensure uniformity in drug content. From day 0 to day 7, all suspensions demonstrated acceptable content levels, with the acceptance value for the first 10 samples of each suspension meeting the L1 criterion (<15). On day 14, all suspensions remained in the acceptable range, except for Dextrose 50%, for which the AV exceeded L1. After 20 additional samples were tested, the final AV also failed to meet the acceptance criterion. A deterioration in content uniformity was observed on day 30, with only Ora-Sweet and Ora-Plus passing the test. This result indicated a noticeable deviation in content uniformity for the Dextrose suspensions. These results further underscored the importance of the consistent distribution of a drug in a compounded suspension, considering the length of storage. Finally, after 60 days, only the Ora-Plus suspension passed the test, whereas all of the other suspensions showed unacceptable content levels, demonstrating the superior stability of the Ora-Plus suspension throughout the study period (Figure 6).

### 2.9. Dissolution Test

**Figure 7:**
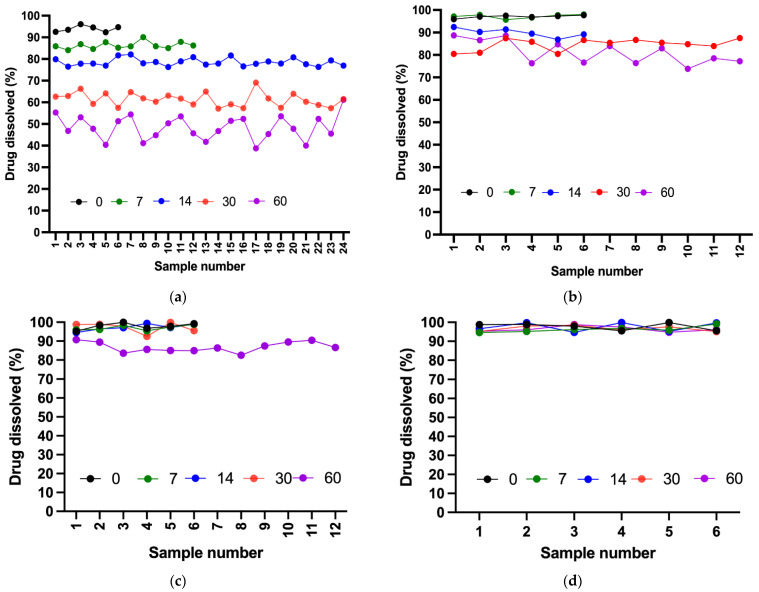
Dissolution profiles of compounded suspensions after 60 min over 60 days: (**a**) Dextrose 50%; (**b**) Dextrose 70%; (**c**) Ora-Sweet; (**d**) Ora-Plus.

All four formulations demonstrated acceptable dissolution profiles on day 0 because all samples passed the Stage 1 (S1) acceptance criteria defined by the USP. By day 7, all suspensions remained stable except for Dextrose 50%, which required additional stage 2 (S2) testing. Although the suspension passed S2, early instability was evident. On day 14, all suspensions passed S1, whereas Dextrose 50% clearly deviated from the expected release levels within 60 min (≥80%) and failed to pass stage 3 (S3) testing. On day 30, Ora-Sweet and Ora-Plus continued to pass S1, and Dextrose 70% proceeded toward and passed S2. By the end of the study (day 60), Dextrose 70% and Ora-Sweet required additional testing (S2) but remained within the established limits, whereas Ora-Plus passed S1, indicating a consistent release rate over the study period. Figure 7 compares the drug release percentages of the four furosemide formulations after 60 min over the study period.

A two-way ANOVA was applied to assess the effect of storage time on the percentage of drug dissolved in the Dextrose 50% (D50%) formulation. The analysis revealed a highly significant effect of time (*p* < 0.0001), indicating that drug dissolution was strongly influenced by storage duration. In contrast, variation across individual samples was not statistically significant (*p* = 0.7122), confirming consistency in sampling and measurement.

Similarly, a two-way ANOVA was conducted to evaluate the effect of storage time on drug dissolution from the Dextrose 70% (D70%) formulation. The results showed a statistically significant impact of time (*p* < 0.0001), confirming that drug dissolution decreased significantly with prolonged storage. However, no significant variation was observed among individual samples (*p* = 0.7982), indicating uniform performance across replicates.

For the Ora-Sweet formulation, a two-way ANOVA also demonstrated a statistically significant effect of storage time on drug dissolution (*p* < 0.0001), suggesting a marked decline in the percentage of drug dissolved with extended storage. In contrast, differences among individual replicates were not statistically significant (*p* = 0.5801), reflecting consistent performance across samples.

Lastly, for the Ora-Plus formulation, the two-way ANOVA showed no statistically significant effect of storage time on drug dissolution (*p* = 0.5677). Additionally, no significant variation was found among individual samples (*p* = 0.8662), confirming the consistency and stability of the formulation over the 60-day period. These findings indicate that drug release from the Ora-Plus vehicle remained stable and uniform throughout the study duration.

### 2.10. Microbiological Stability

None of the samples showed turbidity in either the soybean–casein digest medium or the fluid thioglycolate when incubated at 35 ± 2 °C during the study period. To ensure the absence of microbial growth, additional testing was carried out for samples showing slight turbidity. This was performed by incubating the samples for 48 h after subculturing them in lysogeny broth and Sabouraud Dextrose agar. This further testing yielded no evidence of colony formation, thereby verifying the absence of microbial contamination.

## 3. Discussion

The aim of the present study was to evaluate the stability and performance of furosemide suspensions formulated with various vehicles, namely, Dextrose 50%, Dextrose 70%, Ora-Sweet, and Ora-Plus. The findings revealed significant differences in physicochemical properties, stability, and drug release profiles among the formulations, which are critical for optimizing furosemide’s therapeutic efficacy and patient acceptability. These results align with previous studies that emphasize the importance of vehicle selection in maintaining drug stability and bioavailability [23].

The establishment of a standard calibration curve for furosemide demonstrated a high degree of linearity (R^2^ = 0.999) across the tested concentration range (2–12 µg/mL). This strong correlation supports the reliability of the spectrophotometric method used for quantifying furosemide concentrations in compounded suspensions. The significant *p*-value (<0.0001) further validates the method’s suitability for routine analysis in pharmaceutical formulations. These findings are consistent with earlier research that confirmed the effectiveness of spectrophotometric methods for drug quantification [24].

The visual and organoleptic properties of the suspensions were generally satisfactory, with all formulations being odorless and visually distinct. However, the notable foam formation in the Dextrose 70% suspension by day 7 indicated potential instability, likely due to physicochemical changes. Such changes could negatively affect patient compliance and the overall therapeutic effectiveness of the formulation, corroborating findings from previous studies that noted similar stability issues in high-sugar formulations [25,26]. In contrast, the stability of the visual appearance of the other suspensions suggests that they may be more suitable for long-term storage and patient use.

The pH of the suspensions significantly influences drug solubility, stability, and patient acceptability. The Dextrose 50% suspension exhibited an initial decline in pH followed by stabilization, indicating a relatively favorable environment for drug stability. Conversely, the consistently low pH of the Dextrose 70% suspension raises concerns regarding its suitability as a vehicle, particularly for drugs requiring a neutral pH for optimal stability and solubility. This observation aligns with previous research highlighting the detrimental effects of low pH on drug stability [27]. The Ora-Sweet and Ora-Plus formulations demonstrated superior pH stability, with Ora-Plus showing the most consistent pH values over time, suggesting that it may be the most appropriate vehicle for furosemide suspensions.

Sedimentation profiles indicated that the Dextrose 50% suspension remained stable over time, while the Dextrose 70% suspension’s foam-like appearance hindered further measurements. The lack of sedimentation in the Ora-Sweet and Ora-Plus formulations initially suggests effective suspending agents. Previous studies have similarly reported the effectiveness of these agents in maintaining suspension stability [28]. However, the higher sedimentation volumes observed in Ora-Plus after day 7 indicate potential challenges to maintaining suspension stability over time, corroborating findings from other studies that emphasize the role of viscosity in suspension stability [29].

Viscosity is a critical factor influencing the stability of suspensions. This finding aligns with previous studies that have reported similar viscosity trends among various pharmaceutical suspensions. For instance, Tanninen et al. [28] noted that suspensions formulated with syrups such as Ora-Sweet typically exhibit higher viscosities compared to those prepared with simple sugars or aqueous solutions. This effect is attributed to the thickening agents present in Ora-Sweet.

An important aspect to note in the selection of the suspending vehicle for extemporaneous compounding is the osmolarity parameter, particularly in sensitive populations such as pediatrics and patients with metabolic sensitivity. Dextrose 50% and Dextrose 70% osmolarities are considered extremely high in comparison with physiological osmolarity, Ora-Sweet, and Ora-Plus. The oral administration of Dextrose 50% and Dextrose 70%, especially in pediatric patients or those with compromised gastrointestinal (GI) function, may lead to side effects, including diarrhea and abdominal cramping. These side effects can decrease patient compliance and pose additional risks [30].

In this study, Dextrose 50% and Dextrose 70% were included to reflect real-world scenarios where preferred vehicles (Ora-Sweet and Ora-Plus) may be unavailable due to hospital shortages. However, these vehicles are not recommended for routine compounding. Moreover, their high sugar content renders them unsuitable for diabetic patients, and pharmacists should carefully evaluate individual patient needs before selecting such vehicles [31].

The particle size analysis revealed that the Dextrose 50% and Dextrose 70% suspensions showed initial stability but increased in particle size over time, indicating aggregation. In contrast, the Ora-Sweet formulation maintained a consistent particle size, while Ora-Plus exhibited a marked increase, suggesting a tendency toward sedimentation. These findings underscore the importance of vehicle selection in maintaining particle size consistency, which is crucial for ensuring uniform drug distribution and bioavailability. The zeta potential results further confirmed these observations: Ora-Plus demonstrated the most negative zeta potential values, indicating superior electrostatic repulsion and dispersion stability compared to the other formulations. This characteristic may contribute to the long-term stability of the Ora-Plus suspension, aligning with prior research that highlights the significance of zeta potential in suspension formulations [28].

Content uniformity assessments revealed that only the Ora-Plus suspension consistently met acceptance criteria over the 60-day storage period, highlighting the impact of formulation choice on drug distribution within suspensions. The deterioration in content uniformity for the dextrose-based formulations over time is concerning, as it may lead to inconsistent dosing and therapeutic outcomes. This observation is consistent with previous studies that have reported similar challenges in achieving content uniformity in suspensions [32].

The absence of microbial contamination in all formulations throughout the study is a positive outcome, ensuring the safety and integrity of the compounded suspensions. Microbiological testing underscores the importance of maintaining sterility in compounded formulations, particularly for oral suspensions that may be susceptible to microbial growth. This aspect is crucial for patient safety and the overall efficacy of treatment, aligning with established guidelines in pharmaceutical compounding [33].

The dissolution profiles further illustrate the implications of vehicle selection, with Ora-Sweet and Ora-Plus maintaining acceptable release rates throughout the study, while Dextrose 50% exhibited significant deviations. This supports prior findings on the impact of vehicle choice on drug release rates [23]. Although dissolution testing is primarily applied to solid dosage forms, previous studies have described adaptations for application to oral suspensions. In the current study, we utilized the USP dissolution protocol for furosemide tablets in order to assess the extent to which the compositions of the vehicle impacted the release behavior of furosemide from oral suspensions. This approach is particularly informative for shedding further light on the formulation-dependent availability of a drug over time.

The pH of the suspension influences the chemical stability of furosemide, with deviations potentially accelerating degradation. Sedimentation volume directly affect dose uniformity, which is critical for pediatric patients who require precise dosing to avoid therapeutic failure or toxicity. Content uniformity ensures the consistent delivery of the intended dose, which underpins the formulation’s efficacy. Dissolution behavior serves as a surrogate for bioavailability; reduced dissolution may compromise systemic drug exposure and clinical response. Lastly, microbiological stability is vital for safety, particularly in vulnerable populations such as neonates, where contamination can lead to serious infections. These indicators collectively represent both the therapeutic performance and safety profile of compounded furosemide suspensions.

## 4. Materials and Methods

### 4.1. Materials

The furosemide tablets (Diusemide^®^, 40 mg, APM; Batch No. 356546, Exp. Date: 11/2027) used to conduct stability testing were procured from a private pharmacy. The tablet formulation contains the following excipients: starch, corn, lactose monohydrate, magnesium stearate, and talc. The reference standard, furosemide USP (≥99.5% purity), was obtained from Merck (Rahway, NJ, USA). The vehicles used in this study (Ora-Sweet, Ora-Plus, Dextrose 50%, and Dextrose 70%) were purchased from Medisca (Plattsburgh, NY, USA). Lennox L Broth was procured from Invitrogen (Paisley, UK), and tryptic soya broth (also known as soybean casein digest broth) was obtained from HiMedia (Maharashtra, India). The reference strains included in the microbiological study (*Staphylococcus aureus*, *Pseudomonas aeruginosa*, *Bacillus subtilis*, *Candida albicans*, and *Shigella sonnei*) were purchased from Microbiologics (Saint Cloud, MN, USA). All materials used throughout the study were of analytical grade.

### 4.2. Standard Calibration Curve

A 1000 µg/mL standard stock solution of furosemide was prepared by accurately weighing and dissolving 50 mg of furosemide in 50 mL of 0.1 N NaOH. To determine the wavelength of the maximum absorbance (λ) of furosemide, the standard stock solution was scanned within the ultraviolet (UV)–visible spectrum (200–400 nm) using a UV spectrophotometer. The maximum absorbance peak identified from the absorption curve was at a wavelength of 270 nm, which was set for all measurements conducted in the present study. Standard solutions were created using a series of volumetric flasks to construct the calibration curve using the following method. The stock solution (1000 µg/mL) was diluted to a concentration of 100 µg/mL with 0.1 N NaOH and then to final concentrations of 2, 4, 6, 8, 10, and 12 µg/mL. The possibility of measurement variability was considered, and to ensure data reliability, all concentrations were prepared and assayed in triplicate [34].

### 4.3. Extemporaneous Preparation of Furosemide Oral Suspension

Compounded furosemide oral suspensions (2 mg/mL) were prepared following standard extemporaneous preparation practices commonly applied in hospital pharmacies, in accordance with USP <795> Pharmaceutical Compounding—Nonsterile Preparations [31]. Briefly, five furosemide tablets (0.2 g) were crushed and ground into a fine powder (0.2 g) using a mortar and pestle for two minutes. The resultant powder was then levigated with a small amount of each vehicle individually (Dextrose 50%, Dextrose 70%, Ora-Sweet, or Ora-Plus) to form a paste. This was then further diluted with the vehicle to a final volume of 100 mL, transferred to an amber glass bottle, and stored at 4 °C in a refrigerator for 60 days. For each vehicle, five suspensions were prepared, each designated for a specific time point to minimize handling and prevent variability due to repeated sampling. The suspensions were evaluated immediately (day 0) and on days 7, 14, 30, and 60.

### 4.4. Visual Appearance and Organoleptic Properties of the Compounded Suspensions

At each time point, the compounded suspensions were visually examined for color changes. The samples were transferred into transparent glass beakers and set against a black background. The samples were also examined for changes in odor, ease of redispersion, and signs of caking. The suspension vehicles alone (Dextrose 50%, Dextrose 70%, Ora-Sweet, and Ora-Plus) were used as controls [35].

### 4.5. Changes in pH Values

The pH values of the suspensions were determined (*n* = 3) at each time point with a bench pH/ORP meter (HI 2211, Hanna Instruments, Leighton Buzzard, UK) that had been calibrated before each set of tests using standard buffer solutions (pH 3.00, 4.00, 7.00, and 10.00) [35].

### 4.6. Sedimentation Volume

The sedimentation volume (F), described as the ratio of the ultimate sediment volume (Vu) to the initial (original) volume of the suspension (Vo) [36], was determined (*n* = 3) using the following formula:

F = Vu/Vo(1)

Prior to measurement, each suspension was manually shaken for one minute to ensure uniform dispersion, and then allowed to stand at room temperature for five minutes.

### 4.7. Viscosity Measurements

A Brookfield R/S+ rheometer with a C50-1 cone spindle (Middleboro, MA, USA) was used to measure suspension viscosity (*n* = 3). A sample of the suspension (0.25 g) was placed on the rheometer plate, and the RPM settings were set within the range of 400–1000 at room temperature [35].

### 4.8. Particle Size and Zeta Potential

At room temperature, and after appropriately diluting the suspension samples, the zeta potential, particle size, and polydispersity index were measured (*n* = 3) using photon correlation spectroscopy with a Zetasizer (Nano ZS, Malvern Panalytical, Malvern, UK) [35].

### 4.9. Microbiological Test

The microbiological stability of the compounded suspensions was evaluated in triplicate at each time point using compendial microbial limit testing techniques. Specifically, samples from each suspension were aseptically inoculated into two types of culture media to test for any microbial growth, following pharmacopeial guidelines [29]. Soybean–casein digest broth (tryptic soy broth) was used to support the growth of aerobic bacteria and fungi, and was incubated at 20–25 °C. Fluid thioglycolate broth was used to support the growth of anaerobic or facultative bacteria, and was incubated at 30–35 °C. Each inoculated medium was incubated for at least 14 days and observed periodically for any signs of microbial growth (such as turbidity or sediment). Appropriate controls were included in each test: negative controls (uninoculated media incubated alongside samples) to ensure the media remained sterile, and positive controls (media inoculated with ≤100 colony-forming units of reference organisms) to confirm the capability of the media to support microbial growth. The culture media were qualified by growth promotion tests using standard reference strains—for example, *Bacillus subtilis* and *Staphylococcus aureus* for aerobic bacteria, *Candida albicans* for fungi (in soybean–casein digest medium), and *Clostridium sporogenes* or *Shigella sonnei* for anaerobic bacteria (in fluid thioglycolate medium)—incubated for 3–5 days to verify media suitability [28].

Because the furosemide suspensions were opaque, the direct visual detection of microbial growth in the primary incubation could be unreliable. To address this, after 14 days of incubation, an aliquot (1 mL) from each test container was aseptically transferred into fresh tubes of the same medium and incubated for at least an additional 4 days (a second incubation period) [11]. This subculturing step ensures that even low levels of microorganisms not immediately evident in the turbid suspension can be detected upon further growth in fresh clear media. No microbial growth was concluded for a given sample if no turbidity, pellicle, sediment, or colony formation was observed in any of the test media (in either the initial 14-day incubation or the subsequent subculture) compared to the sterile negative control. All results at each time point were recorded as “growth” or “no growth”, accordingly, with all tested samples showing no detectable microbial growth throughout the 60-day study period [11,37,38].

### 4.10. Content Uniformity Test

A content uniformity test of the suspensions was conducted in accordance with USP recommendations [39]. Prior to sampling, each suspension container was manually shaken for 30 s to ensure homogeneity. A 1 mL aliquot was withdrawn from the middle of the container using a calibrated micropipette. The sample was transferred to a 10 mL volumetric flask, diluted to volume with 0.1 N NaOH, and sonicated for 5 min. Then, 1 mL of this solution was transferred into another 10 mL flask, diluted with 0.1 N NaOH, and sonicated for an additional 2 min. Finally, 1 mL was transferred into a 5 mL flask, diluted to volume with 0.1 N NaOH, and filtered using a 0.45 µm syringe filter before UV spectrophotometric analysis.

For the suspension to pass the test, the acceptance value (AV) of the first 10 samples had to be less than or equal to 15.0 (L1). If the sample exceeded 15.0, the next 20 samples were assayed, and the AV was recalculated. The suspension passed the test if the final AV of the 30 samples was less than or equal to L1 and no individual sample was less than [1 − (0.01) (L2)]M or more than [1 + (0.01) (L2)]M, (L2 = 25).

The following formula was used to determine the AV:

AV = | M − X | + ks(2)

where X is the mean individual content of each dosage unit evaluated, expressed as a percentage of the label claim; M is the reference value; k is an acceptability constant (if *n* = 10, then k = 2.4, and if *n* = 30, then k = 2); and s represents the sample standard deviation [6]. The drug content analysis was conducted using UV spectrophotometry (Libra S22 UV/Vis., Biochrom Ltd., Cambridge, UK) at 270 nm.

### 4.11. Dissolution Test

**Figure 8:**
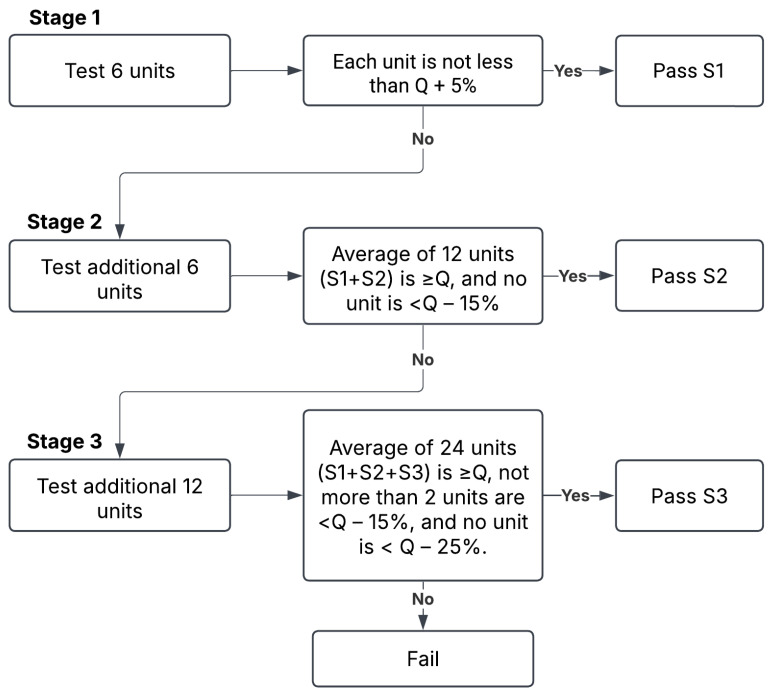
Acceptance criteria for the dissolution of immediate release dosage forms according to USP guidelines.

The dissolution test was performed per the USP monograph for furosemide tablets [39] using USP dissolution apparatus II (PT-DT70, Pharma Test, Hainburg, Germany). The apparatus was set to 50 rpm, and the dissolution medium was 900 mL phosphate buffer (pH 5.8) maintained at 37 ± 0.5 °C. A 5 mL suspension sample was removed and transferred to the dissolution medium vessel. Samples of 5 mL were withdrawn from the zone midway between the dissolving medium surface and the top of the rotating blade at 5, 10, 20, 30, and 60 min intervals and immediately filtered through a 0.22 μm filter to remove any particulate matter. The removed aliquots were immediately replaced with 5 mL of fresh medium at 37 °C to maintain a consistent volume of the medium. The samples were assayed with UV spectrophotometry (Libra S22 UV/Vis., Biochrom Ltd., Cambridge, UK) at a wavelength of 270 nm. The acceptance criteria for dissolution were established according to the USP guidelines for immediate release dosage forms using a three-stage approach (S1, S2, and S3) to determine compliance. The criteria are summarized in Figure 8. Quantity (Q) represents the percentage of the labeled content of the dosage unit that is dissolved at a specified time. For this study, the dissolution specification was set at Q = 80% of the label claim in 60 min, as specified in the furosemide USP monograph [39]. While dissolution testing is primarily used to assess solid dosage forms, it was used in this study to assess the in vitro drug release behavior of furosemide suspensions, and to permit the comparison of furosemide release across the different vehicles.

### 4.12. Stability Study

The prepared furosemide suspensions underwent quality control assessments at multiple time points, as previously outlined [35]. Prior to collecting the samples for analysis, each bottle was shaken by hand for 1 min. The analysis was performed immediately after preparation (day 0) and on days 7, 14, 30, and 60 after being maintained at 4 °C. In each test, three samples (*n* = 3) were collected from the same suspension bottle to assess within-suspension variability. All suspensions were stored and handled in amber glass bottles and under reduced light conditions to minimize the photodegradation of furosemide.

### 4.13. Statistical Analysis

All statistical analyses were conducted using GraphPad Prism 10 statistical software. When appropriate, a two-way analysis of variance (ANOVA) was used to establish statistical significance. A *p*-value of less than 0.05 was considered statistically significant [40].

## 5. Conclusions

In conclusion, the results of this study highlight the significant variability in the stability, physicochemical properties, and performance of furosemide suspensions formulated with different vehicles. The Ora-Plus suspension emerged as the most stable formulation, demonstrating superior pH stability, particle size consistency, zeta potential, content uniformity, and dissolution profiles. These findings underscore the necessity of carefully selecting excipients in compounded formulations to ensure optimal therapeutic outcomes and patient compliance.

## Figures and Tables

**Table 1:** pH measurements of furosemide suspensions prepared using Dextrose 50%, Dextrose 70%, Ora-Sweet, and Ora-Plus vehicles over a 60-day period (*n* = 3).

Formulation Vehicle	Day 0	Day 7	Day 14	Day 30	Day 60
Dextrose 50%	4.57 ± 0.038	4.11 ± 0.010	4.67 ± 0.010	4.68 ± 0.006	4.72 ± 0.006
Dextrose 70%	3.16 ± 0.021	2.93 ± 0.040	3.12 ± 0.006	3.06 ± 0.010	3.06 ± 0.010
Ora-Sweet	4.14 ± 0.010	4.15 ± 0.015	4.20 ± 0.006	4.13 ± 0.012	4.17 ± 0.006
Ora-Plus	4.48 ± 0.010	4.48 ± 0.010	4.47 ± 0.006	4.47 ± 0.006	4.52 ± 0.006

## Data Availability

The original contributions presented in this study are included in the article. Further inquiries can be directed to the corresponding author.

## Supplementary Materials

The following supporting information can be downloaded at: https://www.mdpi.com/article/10.3390/ph18070937/s1, Figure S1: Representative visual appearance of furosemide oral suspensions prepared with different vehicles on Day 7 of storage at 4 °C, protected from light.

## Abbreviations

The following abbreviations are used in this manuscript:

USP	United States Pharmacopeia
BUDs	Beyond use dates
AV	Acceptance value
S	Stage
UV	Ultraviolet
