# Supplementary material for: Stability Assessment of Furosemide Oral Suspension in Hospital Extemporaneous Preparations
Source: Pharmaceuticals (Basel). 2025 Jun 20;18(7):937. doi: 10.3390/ph18070937 (PMC12299591; doi:10.3390/ph18070937)
Supplement: Supplementary file 1 [file pharmaceuticals-18-00937-s001.zip › pharmaceuticals-3657017-supplementary.pdf]

Figure S1. Representative visual appearance of furosemide oral suspensions prepared with different vehicles on Day 7 of storage at 4 °C, protected from light.

Dextrose 50%	Dextrose 70%	Ora-Sweet	Ora-Plus
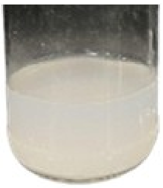	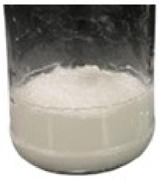	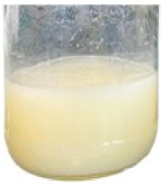	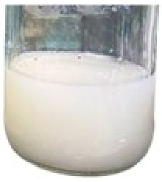
